# Podocyte Markers as Diagnostic Biomarkers of Diabetic Nephropathy: A Case–Control Comparison to Haemato‐Biochemical Markers in a Ghanaian Population

**DOI:** 10.1002/edm2.70240

**Published:** 2026-05-21

**Authors:** Helen Owusu‐Asante, Samuel Nkansah Darko, Ebenezer Senu, Max Annani‐Akollor

**Affiliations:** ^1^ Department of Laboratory Technology Kumasi Technical University Kumasi Ghana; ^2^ Department of Molecular Medicine Kwame Nkrumah University of Science and Technology Kumasi Ghana; ^3^ Department of Clinical Research and Data Science Elite Research and Data Science Institute Kumasi Ashanti Region Ghana; ^4^ Department of Biological Sciences, School of Natural Sciences and Mathematics The University of Texas at Dallas Richardson Texas USA

**Keywords:** biomarkers, diabetic kidney disease, nephrin

## Abstract

**Introduction:**

Several challenges have been raised about the ability of urine microalbumin and eGFR to sufficiently diagnose nephropathy, especially in the early stages. Emerging evidence suggests that podocyte‐associated biomarkers, along with routine haemato‐biochemical parameters, may offer a more accurate and cost‐effective diagnostic alternative. This study, therefore, assessed the performance of podocyte markers and inexpensive haemato‐biochemical parameters in the diagnosis of diabetic nephropathy.

**Methods:**

This unmatched 1:1 case–control study included 134 people living with diabetes (PLWD) as cases and 134 people without diabetes (PwD) as controls. Blood samples were collected from participants for full blood count and separated serum and urine for ELISA quantification of podocyte markers. This study was designed and reported based on the Strengthening the Reporting of Observational Studies in Epidemiology (STROBE) checklist for case–control studies. Statistical analyses were performed using SPSS 26.0 and R Language 4.4.4 at a significant level of 0.05% and 95% confidence interval.

**Results:**

This study found macroalbuminuric PLWD had significantly low mean cell volume, but significantly higher levels of platelet and mean platelet volume. Moreover, nephrin and podocalyxin levels were significantly higher in subjects with microalbuminuria and macroalbuminuria compared to PwD nephropathy subjects. Increasing nephrin levels (AUC = 0.774, *p* = 0.001) and podocalyxin (AUC = 0.744, *p* = 0.001), and decreasing haemoglobin levels (AUC = 0.653, *p* = 0.016) were significantly associated with diabetic nephropathy among PLWD. At a cut‐off ≥ 12.46, nephrin emerged as the best biomarker for detecting diabetic nephropathy with higher sensitivity and specificity.

**Conclusions:**

Urinary podocyte markers, particularly nephrin, together with haematological indices, could provide sensitive yet inexpensive biomarkers for potentially predicting and diagnosing early diabetic nephropathy. Further studies are needed to augment the findings of this study.

## Graphical abstract

This study shows that urinary nephrin and related podocyte injury markers are elevated among people living with diabetes in Ghana, particularly those with macroalbuminuria. Nephrin demonstrated discriminatory ability, supporting its role as an exploratory biomarker for diabetic nephropathy rather than a standalone diagnostic tool.

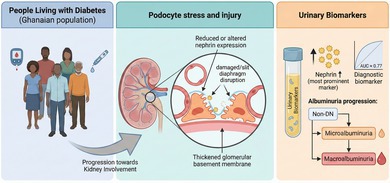

## 1 Introduction

The microvascular complications of diabetes, usually accelerated by sustained hyperglycaemia, are responsible for most of the morbidities associated with the disease. Retinopathy, neuropathy, and nephropathy are the commonest complications, and studies have shown that in the course of the disease, the majority of people with diabetes suffer from one or more of these complications, either overtly or asymptomatically [1]. Diabetic nephropathy is currently the main cause of end‐stage kidney disease, and it is known to be present in about 30% and 40% of Type 1 and Type 2 people living with diabetes (PLWD) respectively [2].

Traditionally, nephropathy has been diagnosed using glomerular filtration rate (GFR); however, this method is limited as many biological factors can affect GFR. Additionally, GFR is unable to detect early kidney damage [3, 4]. Another drawback in the diagnosis of nephropathy is the fact that microalbumin, which used to be the gold standard, is challenged due to significant kidney damage occurring before albuminuria is detectable. Second, some PLWD remain normoalbuminuric despite significant renal damage [5, 6, 7].

Recently, research has increasingly focused on identifying more sensitive biomarkers. Protein‐based markers, including kidney injury molecule‐1 (KIM‐1) and neutrophil gelatinase‐associated lipocalin (NGAL), have improved detection of kidney injury but still lack sufficient specificity and cost‐effectiveness for routine clinical use [8]. In more recent times, advances in high‐throughput technologies have shifted attention towards metabolomics, which enables comprehensive profiling of small‐molecule metabolites in biological samples. Metabolomic analyses provide a dynamic metabolic fingerprint of disease conditions and have led to the identification of novel biomarkers such as acylcarnitines, glycerolipids and tryptophan‐derived metabolites that may improve disease stratification and mechanistic understanding of CKD [9].

Although these novel biomarkers offer the promise of greater diagnostic sensitivity, they remain prohibitively expensive and largely impractical for implementation in developing countries, where health systems are often severely under‐resourced.

In light of these drawbacks, there is a need for more sensitive biomarkers that are better able to predict and detect early kidney disease as well as being inexpensive and readily available to all patients, especially those in resource‐limited settings. Urinary podocyte markers have shown promise in recent times as biomarkers of early detection and prediction of diabetic kidney disease. Two podocyte‐associated proteins, nephrin and podocalyxin, have been investigated lately as possible markers of early kidney disease [10]. Nephrin, encoded by the *NPHSI* gene, is a transmembrane protein in the podocyte foot process that maintains podocyte structure [11]. Nephrin is also found to be present in urine before albuminuria presents and, therefore, can be valuable in predicting and diagnosing kidney disease [12]. Podocalyxin is a transmembrane protein found on the apical regions of the podocytes, which are specialized epithelial cells that line the surface of the glomerular basement membrane. Podocytes carry a negative charge that helps to repel some proteins, such as albumin and globulins, during glomerular filtration [10, 13]. Several studies have also reported podocalyxin as a valuable marker of early kidney disease [12, 13, 14, 15].

Since these podocyte markers promise to be a relatively inexpensive means of predicting and detecting early renal damage, this study assessed their performance in the Ghanaian population in comparison to the known haemato‐biochemical markers of diabetic nephropathy.

## 2 Materials and Methods

### 2.1 Study Design and Study Site

An unmatched 1:1 case–control study was conducted at the Kwadaso Seventh‐Day Adventist (SDA) Hospital, Kumasi. Samples were collected between 1st June 2022 and 31st July 2022. This unmatched case–control study was designed and reported based on the Strengthening the Reporting of Observational Studies in Epidemiology (STROBE) checklist for case–control studies.

### 2.2 Study Population

The study employed convenience sampling to recruit 134 people living with diabetes (PLWD) who attend the Diabetes Clinic of the Kwadaso SDA Hospital and 134 people without diabetes (PwD) from the Kwadaso Estate Community in the city of Kumasi, Ghana, as controls. After data collection and cleaning, diabetic participants were sub‐grouped according to microalbumin status.

### 2.3 Inclusion and Exclusion Criteria

Participants were PLWD clients of the hospital who voluntarily consented to being recruited into the study. However, minors, PLWD who had nephropathy from any cause, as well as those with other known haematological malignancies, were excluded from the study.

### 2.4 Sample Size

The sample size was calculated based on two population mean formula using OpenEpi free software version 3, by considering the following assumptions: 95% confidence level (two‐tailed, α = 0.05), 80% power and the ratio of sample size (cases/control) was 1:1. The calculation was based on the Fleiss 1981 formula with continuity correction to determine the least number of cases and controls to be admitted in the study.

### 2.5 Ethical Consideration

The study was conducted in accordance with the ethical principles as outlined in the Helsinki declaration [16] and consistent with Good Clinical Practice (GCP). The study received ethical approval (reference number CHRPE/AP/75/22) from the Committee on Human Research, Publication and Ethics of the Kwame Nkrumah University of Science and Technology. Informed written consent was obtained from all participants. To ensure patient confidentiality, all patient records were assigned unique identifiers. The authors had no access to information that could identify individual participants during or after data collection.

### 2.6 Sample Analysis and Data Collection

Structured questionnaires were administered to consenting participants to ascertain, among other things, their age, duration of disease, family history of diabetes, and their treatment plan. Blood samples were taken from each participant into ethylenediaminetetraacetic acid (EDTA), fluoride and serum separator tubes for haematological and biochemical assays. Urine samples were collected aseptically into sterile containers. Full blood count was analysed using Sysmex XN 2000 Haematology Analyser (Sysmex Corporation, Japan) and biochemical analysis was done using Mindray BS 200 Biochemical Analyser (Shenzhen Mindray Bio‐Medical Electronics Co. Ltd., China). ELISA was employed to assay serum uromodulin, urine nephrin and urine podocalyxin, with the absorbance measured spectrophotometrically at a wavelength of 450 nm.

### 2.7 Data Analysis

Collected data were entered, cleaned and coded using Microsoft Excel 2021. Statistical analyses and visualization were done using Statistical Package for Social Science (SPSS version 26.0) and R language for statistical computing version 4.0.2. Descriptive statistics were done using frequency and percentage for categorical variables, means and standard deviations for parametric continuous variables, and median and interquartile ranges for nonparametric continuous variables. Normality test for continuous variables was done using the Kolmogorov–Smirnov test. Bar charts were used to illustrate the prevalence and proportions of study variables. Chi‐square test or Fisher's exact test was used to determine the differences and association between categorical variables and study groups. An independent sample *t*‐test was used to compare parametric continuous variables and a Mann–Whitney U‐test was used to compare nonparametric continuous variables between cases and controls. In addition, one‐way ANOVA was used to compare parametric marker levels between three or more study groups, whilst Kruskal–Wallis H‐test was used to compare nonparametric marker levels between three or more study groups, with further post hoc multiple comparison tests by Bonferroni. Density and box and whiskers plot were also used to compare levels of podocyte markers between study groups. A binary and multinomial logistic regression prediction model was used to determine the independent predictors of study variables. Furthermore, the receiver operating characteristics (ROC) analyses were used to assess the diagnostic performance of haematological and podocyte biomarkers for detecting DN among diabetic subjects. Specifically, ROC analyses were performed to discriminate macroalbuminuria from nondiabetic nephropathy (non‐DN) among persons living with diabetes. *p* value < 0.05 and 95% confidence interval were considered statistically significant. All data from this study can be found within the manuscript.

## 3 Results

### 3.1 Sociodemographic and Clinical Characteristics of Study Participants

The mean age of cases (58.76 years) was significantly higher compared to controls (45.94 years; *p* < 0.0001). Cases also had a significantly higher proportion of family history of diabetes (68.2% vs. 33.6%; *p* < 0.0001) compared to controls. The predominant family history of diabetes was from parents (18.9%) and siblings (9.1%) (*p* < 0.0001). Cases had significantly higher BMI (30.27 Kg/m^2^) compared to controls (27.68 Kg/m^2^; *p* = 0.0110) (Table 1).

**TABLE 1:** Sociodemographic and clinical characteristics of study participants.

Variable	Controls (*n* = 134)	Cases (*n* = 134)	*p*
Age (years) (π ± SD)	45.94 ± 15.72	58.76 ± 11.94	**< 0.0001** ^a^
Sex			0.0590^b^
Male	44 (33.6)	31 (23.1)	
Female	87 (66.4)	103 (76.9)	
Marital status			**< 0.0001** ^b^
Single	29 (22.5)	4 (3.1)	
Divorced	5 (3.9)	16 (12.5)	
Widowed	12 (9.3)	37 (28.9)	
Married	83 (64.3)	71 (55.5)	
Educational level			**< 0.0001** ^b^
No formal education	6 (4.6)	22 (16.5)	
Basic	15 (11.5)	73 (54.9)	
Secondary	21 (16.0)	18 (13.5)	
Tertiary	89 (67.9)	20 (15.0)	
Frequency of alcohol intake			**0.0130** ^b^
Never	124 (95.4)	116 (86.6)	
Occasionally	6 (4.6)	18 (13.4)	
Family history of DM	44 (33.6)	90 (68.2)	**< 0.0001** ^b^
Family history of nephropathy	6 (4.6)	7 (5.3)	0.7980^c^
BMI (Kg/m2) (π ± SD)	27.68 ± 6.85	30.27 ± 9.04	**0.0110** ^a^
Systolic blood pressure (π ± SD)	138.51 ± 23.97	140.15 ± 25.21	0.5980^a^
Diastolic blood pressure (π ± SD)	84.77 ± 13.56	81.20 ± 14.41	**0.0450** ^a^

Abbreviations: BMI, body mass index; DM, diabetes mellitus.

^a^ *p* values computed by the independent sample *t*‐test.

^b^ *p* values computed by the Chi‐square test.

^c^ *p* values computed by Fisher's exact test, *p* values < 0.05 and bolded means statistically significant.

### 3.2 Clinical Characteristics of Diabetic Subjects

Among the PLWD subjects, more than half have been diagnosed and have been on treatment for about 5 years (51.9%). The majority were on oral hypoglycaemic agents (81.1%) and a few on insulin (15.9%). In addition, most (64.4%) had mildly decreased eGFR (60–89 mL/min/1.73m^2^), and few had moderately decreased eGFR 45–59 mL/min/1.73m^2^ (7.6%) and severely decreased eGFR > 45 mL/min/1.73m^2^ (2.3%) (Table 2).

**TABLE 2:** Clinical characteristics of diabetic subjects.

Variable	Frequency (*n* = 134)	Percentage (%)
Duration of diabetes (years)		
0–5	68	51.9
6–10	23	8.4
11–15	11	8.4
16–20	11	8.4
≥ 21	18	13.7
Duration of diabetes treatment (years)		
0–5	68	51.9
6–10	23	8.4
11–15	11	8.4
16–20	11	8.4
≥ 21	18	13.7
Type of treatment		
Physical/Diet	4	3.0
Insulin	21	15.9
Oral hypoglycaemic agent	107	81.1
eGFR stage		
Normal	34	25.8
Mild	85	64.4
Moderate	10	7.6
Severe	3	2.3

### 3.3 Haemato‐Biochemical Characteristics of Study Participants

This study found diabetic participants had significantly decreased mean levels of haemoglobin (12.65 g/dL vs. 13.10 g/dL; *p* = 0.0240), mean haematocrit (36.97% vs. 38.82%; *p* = 0.0010), mean red blood cell (RBC) count (4.46 10^6^/μL vs. 4.68 10^6^/μL; *p* = 0.0020), mean platelet distribution width (RDW) (11.59 10^3^/μL vs. 13.48 10^3^/μL; *p* < 0.0001) and mean platelet volume–lymphocyte ratio (MPVLR) (4.95 vs. 5.77; *p* = 0.0010) compared to controls. However, PLWD participants had increased levels of mean white blood cell (WBC) (8.81 10^3^/μL vs. 4.85 10^3^/μL; *p* < 0.0001), mean lymphocytes (2.52 10^3^/μL vs. 2.16; *p* < 0.0001), mean neutrophil (2.67 10^3^/μL vs. 2.05 10^3^/μL; *p* < 0.0001), mean platelet (210.17 10^3^/μL vs. 198.222 10^3^/μL; *p* = 0.0030) and mean lymphocyte monocyte ratio (LMR) (12.40 10^3^/μL vs. 4.98 10^3^/μL; *p* = 0.0010) compared to controls (Table 3). Additionally, PLWD participants had significantly higher mean levels of HDL‐cholesterol (3.15 mmol/L vs. 2.95 mmol/L; *p* = 0.0450), urea (4.41 mmol/L vs. 3.72 mmol/L; *p* < 0.0001), creatinine (80.00 mmol/L vs. 76.00 mmol/L; *p* < 0.0001) and urea–creatinine ratio (25.30 vs. 20.25; p < 0.0001) than compared to controls. Moreover, PLWD in the study had significantly higher mean levels of fasting blood glucose (8.30 mmol/L vs. 5.10 mmol/L; *p* < 0.0001), glycated haemoglobin (7.40% vs. 5.80%; *p* < 0.0001) and urine microalbumin (813.49 mg/g vs. 651.54 mg/g; *p* = 0.0060).

**TABLE 3:** Haemato‐biochemical characteristics of study participants.

Variable	Controls (*n* = 134)	Cases (*n* = 134)	*p*
Haemoglobin (g/dL)^a^	13.10 ± 1.69	12.65 ± 1.47	**0.0240**
Haematocrit (%)^a^	38.82 ± 4.63	36.97 ± 4.27	**0.0010**
RBC (10^6^/μL)^a^	4.68 ± 0.60	4.46 ± 0.51	**0.0020**
MCV (fL)^a^	83.26 ± 6.62	83.24 ± 5.79	0.9790
MCH (pg)^a^	28.07 ± 2.64	28.44 ± 2.49	0.2530
MCHC (g/dL)^a^	33.69 ± 1.47	34.18 ± 1.52	**0.0090**
RDW‐SD (fL)^a^	40.42 ± 3.60	40.19 ± 4.45	0.6430
WBC (10^3^/μL)^a^	4.85 ± 1.58	5.81 ± 1.61	**< 0.0001**
Lymphocytes (10^3^/μL)^a^	2.16 ± 0.64	2.52 ± 0.68	**< 0.0001**
Monocytes (10^3^/μL)^a^	0.46 ± 0.14	0.64 ± 2.00	0.3040
Neutrophil (10^3^/μL)^a^	2.05 ± 1.14	2.67 ± 1.28	**< 0.0001**
Eosinophil (10^3^/μL)^a^	0.12 ± 0.09	0.12 ± 0.12	0.9950
Basophil (10^3^/μL)^a^	0.03 ± 0.01	0.03 ± 0.01	0.1030
Platelet (10^3^/μL)^a^	198.22 ± 70.67	210.17 ± 87.18	**0.0030**
MPV (fL)^a^	11.26 ± 0.94	11.59 ± 1.81	0.0790
PDW (fL)^a^	13.48 ± 2.41	11.59 ± 1.81	**< 0.0001**
LMR^a^	4.98 ± 1.73	12.40 ± 2.02	**0.0010**
NLR^a^	1.00 ± 0.05	1.14 ± 0.68	0.1420
PLR^a^	99.74 ± 51.72	97.70 ± 48.54	0.7430
MPVLR^a^	5.77 ± 2.06	4.95 ± 1.76	**0.0010**
Total cholesterol (mmol/L)^b^	5.04 (4.21–5.83)	5.18 (4.48–6.17)	**0.1810**
Triglyceride (mmol/L)^b^	2.08 (1.22–2.24)	2.12 (1.46–2.78)	**0.0870**
HDL‐cholesterol (mmol/L)^b^	1.18 (0.99–1.41)	1.19 (0.96–1.36)	**0.6850**
LDL‐cholesterol (mmol/L)^b^	2.95 (2.32–3.48)	3.15 (2.51–3.80)	**0.0450**
VLDL‐cholesterol (mmol/L)^b^	0.94 (0.56–1.02)	0.96 (0.65–1.24)	**0.1420**
Urea (mmol/L)^b^	3.72 (3.08–4.47)	4.41 (3.33–6.00)	**< 0.0001**
Creatinine (mmol/L)^b^	76.00 (67.75–86.00)	80.00 (72.00–89.00)	**< 0.0001**
Urea–creatinine ratio^b^	20.25 (12.70–26.53)	25.30 (19.98–34.03)	**< 0.0001**
Fasting blood glucose (mmol/L)^b^	5.10 (4.80–5.70)	8.30 (6.20–10.20)	**< 0.0001**
Glycated haemoglobin (%)^b^	5.80 (5.50–6.33)	7.40 (6.45–8.20)	**< 0.0001**
Urine microalbumin (mg/g)^b^	651.54 (63.46–1038.20)	813.49 (211.53–1418.23)	**0.0060**

^a^ Variables presented as mean ± SD.

^b^ Variables presented as median (IQR), *p* values computed by independent sample *t*‐test and Mann–Whitney U‐Test, *p* values < 0.05 and bolded means statistically significant. MCV = mean cell volume, MCH = mean cell haemoglobin, MCHC = mean cell haemoglobin concentration, mean platelet volume, platelet distribution width, lymphocyte–monocyte ratio, neutrophil–lymphocyte ratio, platelet–lymphocyte ratio, mean platelet volume–lymphocyte ratio.

### 3.4 Comparison of Podocyte Markers Between Cases and Controls

**FIGURE 1:**
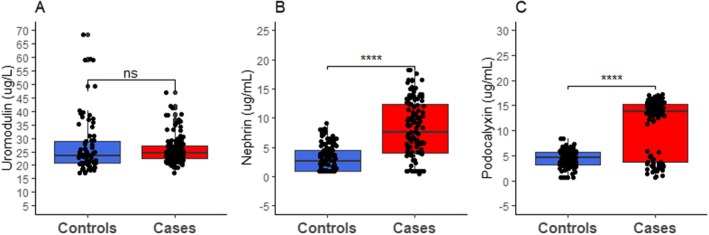
Box and whiskers plot depicting the comparison of podocyte markers between cases and controls. *p* values computed by Mann–Whitney U‐test, ns: Not significant, *p < 0.05, **p < 0.01, ***p < 0.001, ****p < 0.0001.

This study recorded diabetic participants having significantly higher levels of nephrin (*p* < 0.0001) and podocalyxin (*p* < 0.0001) compared to nondiabetic controls. However, the difference in uromodulin between PLWD and controls was not statistically significant (*p* > 0.05) (Figure 1).

### 3.5 Prevalence of Diabetic Nephropathy Among People Living With Type 2 Diabetes

**FIGURE 2:**
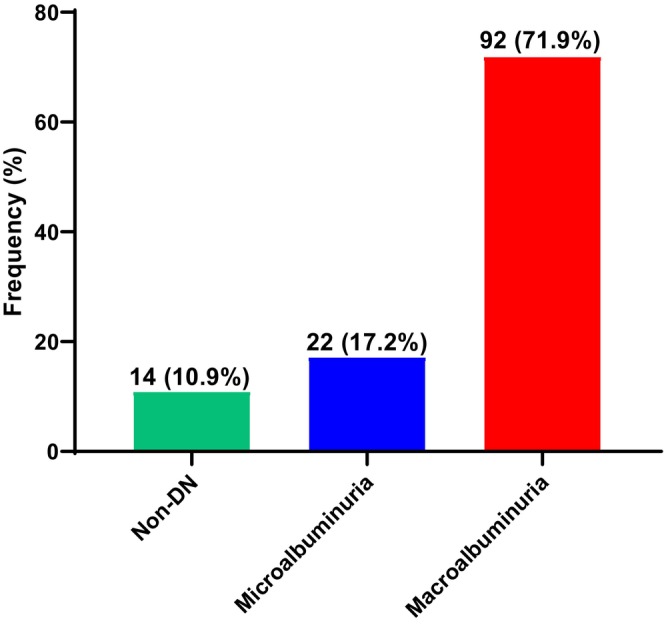
Prevalence of diabetic nephropathy among people living with type 2 diabetes.

This study found that 92 diabetic participants had macroalbuminuria, representing 71.9%, 22 had microalbuminuria, representing 17.2%, whilst 14 (10.9%) were normoalbuminuric (Figure 2).

### 3.6 Haematological and Biochemical Characteristics of Cases With and Without Diabetic Nephropathy

There were significant differences in MCV (*p* = 0.009), platelets (*p* = 0.030), and MPV (*p* = 0.009) between the study groups. In a post hoc multiple comparison test, PLWD with macroalbuminuria had significantly lower MCV (82.37 fL vs. 86.50 fL; *p* < 0.05) compared to those with microalbuminuria. Moreover, people having diabetes with macroalbuminuria had significantly higher levels of platelets (240.09 10^3^/μL vs. 186.58 10^3^/μL; *p* < 0.05), MPV (11.93 fL vs. 10.55 fL; *p* < 0.05) (Table 4). However, this study did not find any significant differences in total cholesterol (*p* = 0.5830), triglyceride (*p* = 0.4870), HDL‐cholesterol (*p* = 0.4210), LDL‐cholesterol (*p* = 0.8870), and VLDL cholesterol (*p* = 0.5650) between the study groups. Moreover, there were no significant differences in urea (*p* = 0.5040), creatinine (*p* = 0.1810), urea–creatinine ratio (*p* = 0.7310), fasting blood glucose (*p* = 0.900) and glycated haemoglobin (*p* = 0.9370) between the study groups (Table 5).

**TABLE 4:** Haematological characteristics of PLWD with and without DN.

Variable	Total (*n* = 128)	Non‐DN (*n* = 14)	Microalbuminuria (*n* = 22)	Macroalbuminuria (*n* = 92)	*p*
Haemoglobin (g/dL)	12.65 ± 1.47	12.99 ± 0.94	13.13 ± 1.61	12.65 ± 1.50	0.122
Haematocrit (%)	36.97 ± 4.27	35.66 ± 2.54	38.20 ± 4.74	36.95 ± 4.36	0.225
RBC (10^6^/μL)	4.46 ± 0.51	4.24 ± 0.42	4.42 ± 0.54	4.51 ± 0.52	0.179
MCV (fL)	83.24 ± 5.79	84.42 ± 5.41	86.50 ± 4.32	82.37 ± 5.93	**0.009** ^a^
MCH (pg)	28.44 ± 2.49	28.64 ± 2.45	29.60 ± 1.89	28.16 ± 2.60	0.058
MCHC (g/dL)	34.18 ± 1.52	33.90 ± 1.17	34.41 ± 1.38	34.16 ± 1.60	0.625
RDW‐SD (fL)	40.19 ± 4.45	39.49 ± 2.67	40.13 ± 4.86	40.22 ± 4.59	0.849
WBC (10^3^/μL)	5.81 ± 1.61	5.75 ± 1.60	6.48 ± 1.69	5.76 ± 1.79	0.792
Lymphocytes (10^3^/μL)	2.52 ± 0.68	2.71 ± 0.64	2.42 ± 0.75	2.49 ± 0.67	0.450
Monocytes (10^3^/μL)	0.64 ± 2.00	0.44 ± 0.14	0.48 ± 0.16	0.73 ± 2.41	0.815
Neutrophil (10^3^/μL)	2.67 ± 1.28	2.48 ± 1.32	2.93 ± 1.37	2.63 ± 1.27	0.553
Eosinophil (10^3^/μL)	0.12 ± 0.12	0.08 ± 0.08	0.15 ± 0.17	0.12 ± 0.11	0.268
Basophil (10^3^/μL)	0.03 ± 0.01	0.03 ± 0.02	0.02 ± 0.01	0.03 ± 0.01	0.776
Platelet (10^3^/μL)	226.58 ± 83.18	186.57 ± 97.88	203.76 ± 80.94	240.09 ± 80.64	**0.030** ^b^
MPV (fL)	11.59 ± 1.81	10.55 ± 1.13	11.01 ± 1.13	11.93 ± 1.96	**0.009** ^b^
PDW (fL)	12.40 ± 1.96	12.15 ± 2.59	12.54 ± 2.27	12.40 ± 1.81	0.855
LMR	5.78 ± 2.02	6.44 ± 1.88	5.32 ± 1.67	5.69 ± 2.12	0.281
NLR	1.14 ± 0.68	0.93 ± 0.40	1.37 ± 0.87	1.13 ± 0.67	0.165
PLR	97.70 ± 48.54	75.30 ± 47.36	97.46 ± 61.36	102.92 ± 45.57	0.147
MPVLR	4.95 ± 1.76	4.12 ± 1.09	5.07 ± 2.01	5.11 ± 1.77	0.171

*Note:* Data presented as means and standard deviations. DN: diabetic nephropathy, *p* values computed by one‐way ANOVA and post hoc multiple comparison by the Bonferroni test.

^a^ Significant difference between microalbuminuria and macroalbuminuria, *p* values < 0.05 and bolded means statistically significant. Despite the Bonferroni adjustment applied to selected comparisons, the large number of univariate analyses increases the risk of type I error. Therefore, statistically significant findings, particularly those with modest *p* values, should be interpreted as hypothesis‐generating rather than confirmatory.

^b^ Significant difference between non‐DN and macroalbuminuria.

**TABLE 5:** Biochemical characteristics of PLWD with and without DN.

Variable	Total (*n* = 132)	Non‐DN (*n* = 14)	Microalbuminuria (*n* = 22)	Macroalbuminuria (*n* = 92)	*p*
Total cholesterol (mmol/L)	5.18 (4.47–6.17)	5.06 (4.81–6.21)	4.92 (4.45–5.65)	5.28 (4.39–6.31)	0.5830
Triglyceride (mmol/L)	2.12 (1.46–2.78)	2.22 (1.16–2.84)	2.01 (1.22–2.42)	2.13 (1.59–2.86)	0.4870
HDL‐cholesterol (mmol/L)	1.19 (0.96–1.36)	1.28 (0.99–1.43)	1.05 (0.87–1.30)	1.18 (0.96–1.38)	0.4210
LDL‐cholesterol (mmol/L)	3.15 (2.51–3.80)	3.32 (2.36–3.64)	3.09 (2.60–3.59)	3.12 (2.43–3.99)	0.8870
VLDL‐cholesterol (mmol/L)	0.96 (0.65–1.24)	1.01 (0.53–1.29)	0.91 (0.56–1.10)	0.96 (0.70–1.29)	0.5650
Urea (mmol/L)	4.41 (3.33–6.01)	3.77 (3.19–5.26)	4.45 (3.71–5.98)	4.36 (3.18–6.03)	0.5040
Creatinine (mmol/L)	80.00 (72.00–89.00)	74.00 (69.75–79.25)	79.00 (72.00–89.00)	82.00 (72.00–89.00)	0.1810
Urea–creatinine ratio	25.80 (19.98–34.03)	22.85 (18.48–37.25)	27.55 (20.10–32.45)	25.80 (19.60–35.50)	0.7310
Fasting blood glucose (mmol/L)	8.30 (6.20–10.20)	7.45 (6.10–10.80)	8.40 (5.60–11.63)	8.30 (6.35–10.28)	0.9000
Glycated haemoglobin (%)	7.40 (6.45–8.20)	7.75 (6.18–8.38)	7.30 (6.40–8.25)	7.30 (6.43–8.10)	0.9370

*Note:* Data presented as median and interquartile ranges. DN: diabetic nephropathy, *p* values computed by one‐way ANOVA, *p* values < 0.05 and bolded means statistically significant.

### 3.7 Comparison of Podocyte Markers Among PLWD With and Without Diabetic Nephropathy

**FIGURE 3:**
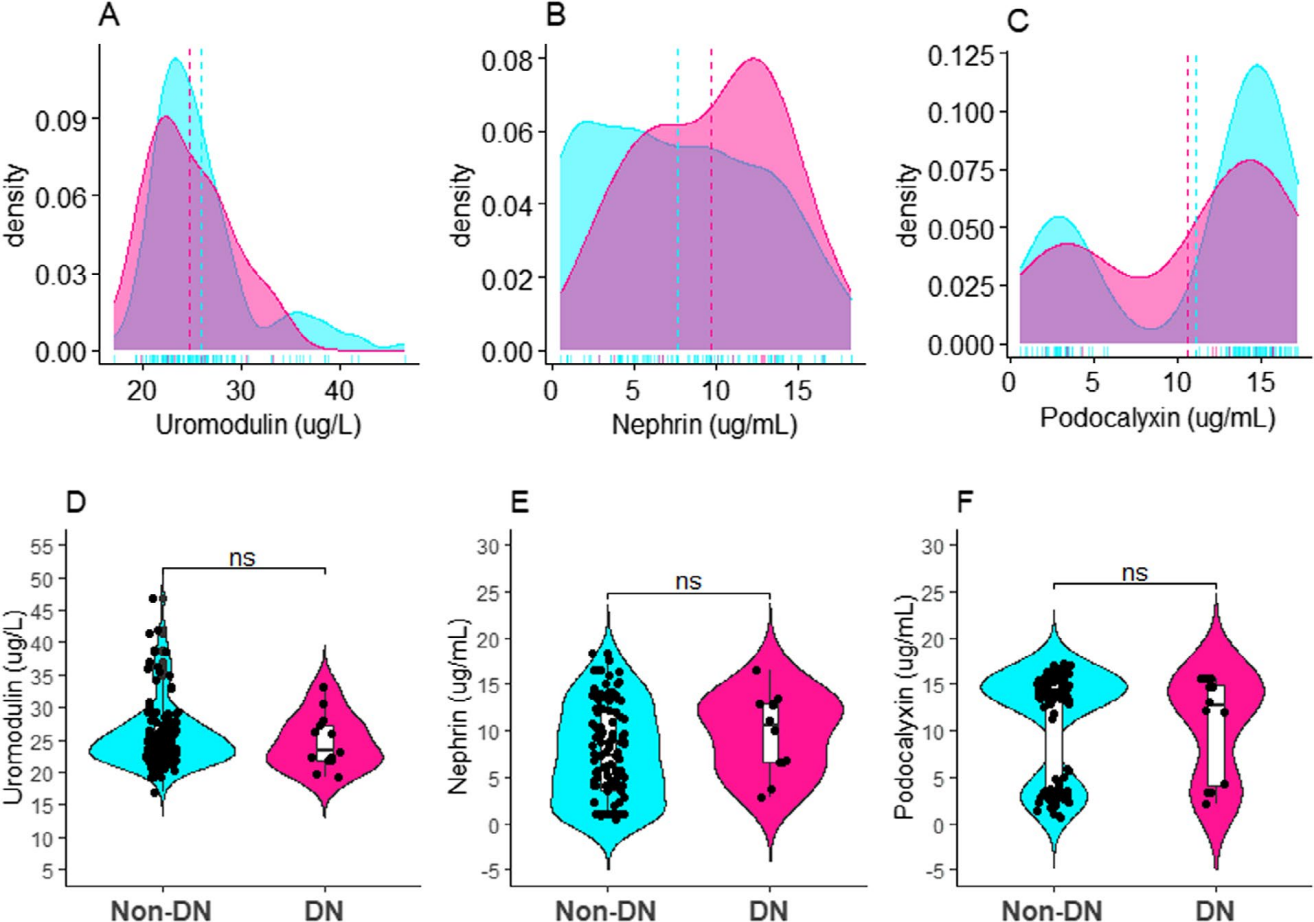
Density (A–C) and box and whiskers plot (D–F) depicting comparison of podocyte markers among diabetic subjects with and without DN. DN, diabetic nephropathy, *p* values computed by Kruskal–Wallis H‐test and post hoc multiple comparison by Bonferroni test, ns: not significant, *p < 0.05, **p < 0.01, ***p < 0.001, ****p < 0.0001.

Nephrin levels were significantly higher in participants with macroalbuminuria (*p* < 0.01) and microalbuminuria (*p* < 0.01) compared to nondiabetic nephropathy participants. Moreover, podocalyxin levels were significantly higher in participants with macroalbuminuria (*p* < 0.01) and microalbuminuria (*p* < 0.01) compared to nondiabetic nephropathy participants (Figure 3).

### 3.8 Association Between Haematological Parameters and DN Among PLWD

Multinomial logistic regression prediction model showed that increasing levels of platelet (*p* = 0.026), MPV (*p* = 0.011), PLR (*p* = 0.048) and MPVLR (*p* = 0.046) were significantly related to an increased likelihood of diabetic nephropathy among PLWD (Table 6).

**TABLE 6:** Association between haematological parameters and DN among PLWD.

Variable	Microalbuminuria (*n* = 22)		Macroalbuminuria (*n* = 92)	*p*
	cOR (95% CI)	*p*	cOR (95% CI)	
Haemoglobin (g/dL)	1.63 (1.02–2.62)	**0.043**	0.95 (0.89–1.84)	0.188
Haematocrit (%)	1.15 (0.98–1.35)	0.087	1.07 (0.94–1.21)	0.299
RBC (10^6^/μL)	1.80 (0.53–6.19)	0.349	2.57 (0.91–7.24)	0.074
MCV (fL)	1.09 (0.95–1.24)	0.238	0.93 (0.84–1.04)	0.215
MCH (pg)	1.21 (0.89–1.64)	0.220	0.92 (0.72–1.17)	0.500
MCHC (g/dL)	1.25 (0.79–1.97)	0.337	1.13 (0.77–1.66)	0.550
RDW‐SD (fL)	1.05 (0.87–1.26)	0.641	1.05 (0.89–1.23)	0.562
WBC (10^3^/μL)	1.10 (0.73–1.66)	0.656	1.00 (0.70–1.41)	0.977
Lymphocytes (10^3^/μL)	0.54 (0.20–1.46)	0.226	0.63 (0.28–1.42)	0.267
Monocytes (10^3^/μL)	3.14 (0.06–178.80)	0.579	3.66 (0.07–194.46)	0.523
Neutrophil (10^3^/μL)	1.30 (0.75–2.26)	0.347	1.11 (0.68–1.82)	0.675
Eosinophil (10^3^/μL)	5825.98 (0.14–24518.48)	0.110	1301.24 (0.04–38967.13)	0.173
Basophil (10^3^/μL)	4.27 (0.03–639.70)	0.474	5.56 (0.05–611.60)	0.603
Platelet (10^3^/μL)	1.00 (0.99–1.01)	0.521	1.01 (1.00–1.02)	**0.026**
MPV (fL)	1.47 (0.76–2.85)	0.256	2.21 (1.20–4.07)	**0.011**
PDW (fL)	1.11 (0.77–1.60)	0.572	1.07 (0.78–1.47)	0.661
LMR	0.77 (0.55–1.07)	0.118	0.84 (0.65–1.10)	0.209
NLR	3.14 (0.80–12.38)	0.102	2.10 (0.57–7.69)	0.262
PLR	1.01 (0.99–1.03)	0.131	1.02 (1.00–1.03)	**0.048**
MPVLR	1.84 (0.97–3.51)	0.064	1.86 (1.01–3.43)	**0.046**

*Note:* Data presented as median and interquartile ranges. *p* values computed by logistic regression prediction model, *p* values < 0.05 and bolded means statistically significant. ORs are exploratory due to the limited sample size in the reference group.

Abbreviations: CI, confidence interval; cOR, crude odds ratio; DN, diabetic nephropathy.

### 3.9 Association Between Biochemical Parameters and DN Among PLWD

In a multinomial logistics regression prediction model, increasing levels of urea (cOR: 1.14, 95% CI (0.86–1.52); *p* = 0.356), creatinine (cOR: 1.05, 95% CI (0.99–1.10); *p* = 0.070), urea–creatinine ratio (cOR: 1.02, 95% CI (0.96–1.08); *p* = 0.524) and fasting blood glucose (cOR: 1.03, 95% CI (0.86–1.23); *p* = 0.781) were insignificantly associated with increased likelihood of macroalbuminuria among PLWD (Table 7).

**TABLE 7:** Association between biochemical parameters and DN among PLWD.

Variable	Microalbuminuria (*n* = 22)		Macroalbuminuria (*n* = 92)	*p*
	cOR (95% CI)	*p*	cOR (95% CI)	
Total cholesterol (mmol/L)	0.85 (0.54–1.35)	0.4950	0.99 (0.68–1.43)	0.948
Triglyceride (mmol/L)	0.67 (0.29–1.54)	0.3440	1.05 (0.54–2.05)	0.881
HDL‐cholesterol (mmol/L)	0.32 (0.05–2.24)	0.2490	0.54 (0.11–2.63)	0.445
LDL‐cholesterol (mmol/L)	0.80 (0.52–1.22)	0.2970	0.86 (0.69–1.07)	0.164
VLDL‐cholesterol (mmol/L)	0.43 (0.07–2.67)	0.3510	1.04 (0.24–4.40)	0.963
Urea (mmol/L)	1.17 (0.85–1.60)	0.3400	1.14 (0.86–1.52)	0.356
Creatinine (mmol/L)	1.05 (0.99–1.11)	0.1180	1.05 (0.99–1.10)	0.070
Urea–creatinine ratio	1.02 (0.95–1.09)	0.5400	1.02 (0.96–1.08)	0.524
Fasting blood glucose (mmol/L)	1.01 (0.82–1.26)	0.9090	1.03 (0.86–1.23)	0.781
Glycated haemoglobin (%)	0.91 (0.52–1.59)	0.7380	0.99 (0.62–1.58)	0.965
Urine microalbumin (mmol/L)	5.90 (0.96–361.10)	0.9130	10.16 (0.90–114.60)	0.902

*Note:* Data presented as median and interquartile ranges. *p* values computed by logistic regression prediction model, *p* values < 0.05 and bolded means statistically significant.

Abbreviations: CI, confidence interval; cOR, Crude odds ratio; DN, Diabetic nephropathy.

### 3.10 Association Between Podocyte Markers and DN Among PLWD

Multinomial logistic regression prediction model showed that increasing levels of nephrin (cOR: 1.31, 95% CI (1.06–1.63); *p* = 0.013) were significantly associated with increased likelihood of microalbuminuria among PLWD. Moreover, increasing levels of nephrin (cOR: 1.32, 95% CI (1.11–1.58); *p* = 0.002) and podocalyxin (cOR: 1.70, 95% CI (1.06–1.10‐2.62); *p* = 0.017) were significantly associated with increased likelihood of macroalbuminuria among PLWD.

In the adjustment logistics regression model, after adjusting for age, sex and other possible confounders, increasing nephrin levels (aOR: 1.35, 95% CI (1.04–1.76); *p* = 0.023) were independently associated with increased likelihood of microalbuminuria among PLWD. Similarly, increasing levels of nephrin (aOR: 1.41, 95% CI (1.11–1.78); *p* = 0.004) and podocalyxin (aOR: 1.84, 95% CI (1.06–1.09–3.12); *p* = 0.012) were independently associated with increased likelihood of macroalbuminuria among PLWD (Table 8).

**TABLE 8:** Association of podocyte markers with diabetic nephropathy among people living with diabetes.

Variable	Microalbuminuria (*n* = 22)		Macroalbuminuria (*n* = 92)	*p*
	cOR (95% CI)	*p*	cOR (95% CI)	
Uromodulin (μg/L)	0.94 (0.78–1.13)	0.488	1.01 (0.88–1.17)	0.847
Nephrin (μg/mL)	1.31 (1.06–1.63)	**0.013**	1.32 (1.11–1.58)	**0.002**
Podocalyxin (μg/mL)	1.58 (0.97–2.57)	0.068	1.70 (1.10–2.62)	**0.017**

Variable	aOR (95% CI)	*p*	aOR (95% CI)	*p*
Uromodulin (μg/L)	0.88 (0.72–1.08)	0.229	0.98 (0.85–1.14)	0.787
Nephrin (μg/mL)	1.35 (1.04–1.76)	**0.023**	1.41 (1.11–1.78)	**0.004**
Podocalyxin (μg/mL)	1.82 (0.97–3.43)	0.064	1.84 (1.09–3.12)	**0.024**

*Note:* DN: diabetic nephropathy, cOR: crude odds ratio, aOR: adjusted odds ratio, CI: confidence interval, *p* values computed by multinomial logistic regression prediction model, model adjusted for age, sex, blood pressure, body mass index, glycaemic control, duration or diagnosis and treatment, *p* values < 0.05 and bolded means statistically significant.

### 3.11 Diagnostic Performance of Podocyte Biomarkers as a Diagnostic Marker for Diabetic Nephropathy Among Diabetic Participants

**FIGURE 4:**
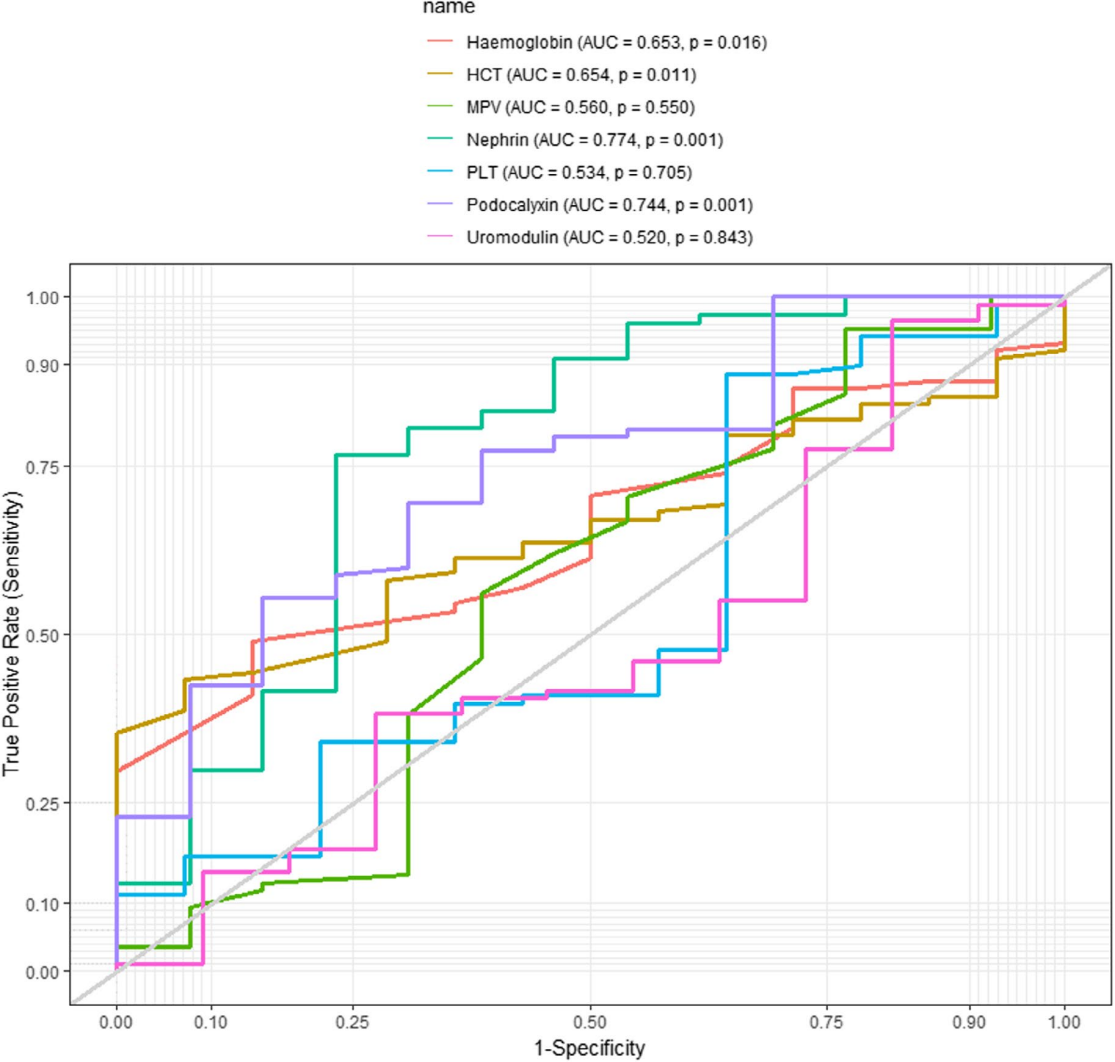
Receiver operating characteristics (ROC) curves depicting the diagnostic performance of podocyte biomarkers for detecting diabetic nephropathy among PLWD. ROC curves depicting discrimination of macroalbuminuria versus non‐DN diabetic participants. AUC, area under the curve.

The receiver operator characteristics (ROC) analysis was used to evaluate the diagnostic performance of podocyte biomarkers in detecting diabetic nephropathy among PLWD. Although haemoglobin, nephrin, and podocalyxin showed significant results, increasing nephrin levels (AUC = 0.774, *p* = 0.001) emerged as the best diagnostic marker of diabetic nephropathy (Figure 4).

At a cut‐off of ≥ 12.46, nephrin was the best biomarker for detecting diabetic nephropathy in the advanced stage using macroalbuminuria among PLWD with a sensitivity of 76.6%, a specificity of 76.9%, an area under the curve of 77.4% and an accuracy of 76.7. For podocalyxin, a cut‐off of ≤ 13.94, could detect diabetic nephropathy among PLWD with a sensitivity of 55.4%, specificity of 84.6%, area under the curve and accuracy of 77.4% and 59.0% respectively. With platelets, a cut‐off of ≥ 112.00, could detect diabetic nephropathy among PLWD with a sensitivity of 88.6%, specificity of 35.7%, area under the curve and accuracy of 53.4% and 81.4% respectively (Table 9).

**TABLE 9:** Diagnostic performance of podocyte biomarkers for detecting DN among PLWD.

Marker	Cut‐off	Sensitivity (95% CI)	Specificity (95% CI)	PPV	NPV	LR+	LR‐	Accuracy
Uromodulin (μg/L)	≥ 24.204	44.9 (35.0–55.3)	72.7 (42.8–90.5)	93.0	14.0	1.65	0.76	48.0
Nephrin (μg/mL)	**≥ 12.46**	**76.6 (65.9–84.7)**	**76.9 (48.9–92.2)**	**95.2**	**35.7**	**3.32**	**0.30**	**76.7**
Podocalyxin (μg/mL)	≤ 13.94	55.4 (45.3–65.2)	84.6 (56.3–96.6)	96.2	21.2	3.60	0.53	59.0
Haemoglobin (g/dL)	≤ 13.00	48.9 (38.7–59.1)	85.7 (58.6–97.0)	95.6	21.1	3.42	0.60	53.9
Haematocrit (%)	≥ 38.20	43.2 (33.3–53.6)	92.9 (66.1–100.0)	97.4	20.6	6.05	0.61	50.0
Platelet (10^3^/μL)	≥ 112.00	88.6 (80.1–93.8)	35.7 (16.4–61.4)	89.7	33.3	1.38	0.32	81.4
Mean platelet volume (fL)	≥ 9.50	95.2 (87.9–98.4)	23.1 (7.8–51.1)	88.9	42.9	1.24	0.21	85.6

*Note:* At a cut‐off of ≥ 12.46, nephrin was the best biomarker for detecting diabetic nephropathy with a sensitivity of 76.6%, specificity of 76.9%, area under the curve of 77.4% and an accuracy of 76.7.

Abbreviations: CI, confidence interval; LR−, negative likelihood ratio; LR+, positive likelihood ratio; NPV, negative predictive value; PPV, positive predictive value.

**FIGURE 5:**
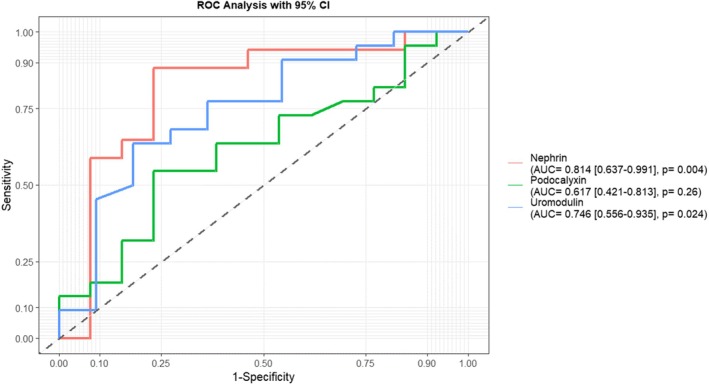
Receiver operating characteristics (ROC) curves depicting the diagnostic performance of podocyte biomarkers for detecting early diabetic nephropathy among PLWD. ROC curves depicting discrimination of microalbuminuria versus non‐DN diabetic participants. AUC, area under the curve.

More importantly, nephrin was the best biomarker for early‐stage detection of diabetic nephropathy using microalbuminuria among PLWD with an area under the curve of 81.4% (95% CI: 0.637–0.991; *p* = 0.004). For uromodulin, a cut‐off could detect diabetic nephropathy using microalbuminuria among PLWD with an area under the curve of 74.6% (95% CI: 0.556–0.935, *p* = 0.024) (Figure 5).

**FIGURE 6:**
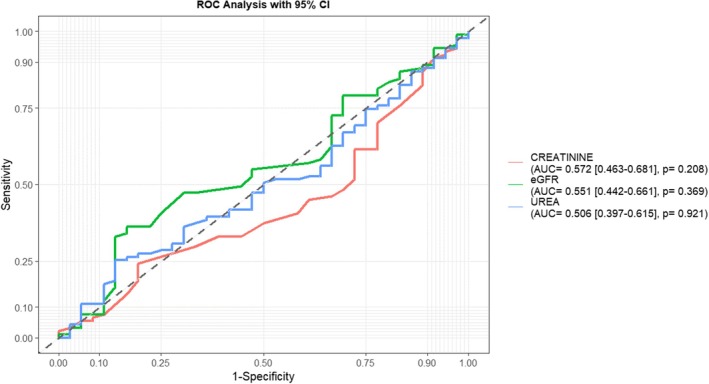
Receiver operating characteristics (ROC) curves depicting diagnostic performance of traditional biomarkers (creatinine, urea and eGFR) for detecting diabetic nephropathy among PLWD. ROC curves depicting discrimination of macroalbuminuria versus non‐DN diabetic participants. AUC, area under the curve.

However, traditional biomarkers such as creatinine, urea and eGFR had low diagnostic performances for detecting diabetic nephropathy among PLWD (Figure 6).

## 4 Discussion

The study has shown that among this Ghanaian diabetic cohort, decreasing haemoglobin concentration as well as increasing urinary nephrin and podocalyxin are significant biomarkers of diabetic nephropathy.

Expectedly, PLWD in this study were hyperglycaemic compared to controls and had higher serum urea and creatinine levels. The deleterious effect of uncontrolled blood glucose includes renal dysfunction, which, if not properly managed, can eventually cause end‐stage renal disease [16, 17]. This study employed the use of the urine albumin to creatinine ratio to report a diabetic kidney disease prevalence of 89.1%. This figure is much higher than what has been reported in Ghana earlier [18], however, that study based its calculation on eGFR using the CKD‐EPI formula, which is a possible reason for the differences.

The consequences of long‐term hyperglycaemia have been implicated in the decreased red blood cell counts in PLWD. Hyperglycaemia induces an elevation in reactive oxygen species, which in turn causes red blood cells to become more rigid. This can impact the red blood cells' flexibility and ability to move through capillaries, potentially leading to vascular problems as well as a reduction in their lifespan [19]. Diabetes also affects red blood cells both quantitatively and qualitatively, altering both the size and shape of RBCs and perhaps disrupting their functional characteristics [20, 21]. Hyperglycaemia also causes distortions in the cell membrane and the activities of some membrane transporters, leading to a shorter lifespan [22].

Blood cell parameters have been reported to be significantly impaired in PLWD compared to PwD, a fact corroborated by the findings of this study. Among the diabetic cases, red cell count, haemoglobin, and haematocrit are reduced, similar to the report by Adane et al. [23, 24, 25]. Hyperglycaemia, with its resultant increased red cell glycation, causes the red cells to become more rigid and less deformable. This increases the removal of the cells from circulation and thus a decrease in haematological parameters. In agreement with previous studies, mean cell haemoglobin concentration [26], white blood cells [27, 28] and platelets [29, 30] are raised. The chronic inflammation associated with hyperglycaemia causes the observed changes in white blood cell count and function, as well as platelet hyperactivity. These changes in white cells and platelets are representative of the immune and vascular complications associated with poor glycaemic control.

This study found that mean cell volume could be utilized as a marker for renal damage, as it was significantly reduced as PLWD progressed from microalbuminuria to macroalbuminuria. This agrees with reports by Nombwende [31] and Anandhasayanam [32]. These changes in the red blood cells are indicative of the deleterious effect of proteinuria on the erythrocytes [33]. This could possibly be due to a reduction in erythropoietin from the kidneys as renal damage increases and subsequently impaired haemopoiesis, as well as the accumulation of uraemic toxins, which affect red cell lifespan and production.

Additionally, decreasing haemoglobin was found to be significantly associated with kidney disease, with an AUC of 0.653. This finding corroborates reports by other researchers, such as Chen [34] and Okada [35], who also reported haemoglobin concentration to be negatively associated with urinary albumin to creatinine ratio. In PLWD with kidney disease, red blood cells undergo severe morphological and functional alterations, which ultimately lead to their shortened lifespan in circulation [24, 36]. Bissinger [33] asserted that the shortened lifespan is a result of the expression of phosphatidylserine on the red cell surface, which made them more susceptible to clearance by immune cells. Moreover, platelet count and mean platelet volume also increased significantly as PLWD developed kidney dysfunction, and at a cut‐off of ≥ 112.00, platelets could detect macroalbuminuria among diabetic subjects with a sensitivity of 88.6%. Platelet indices have been reported to be biomarkers of kidney disease by several authors [34, 35, 36, 37, 38, 39]. For years, the role of platelets in the complications associated with kidney disease has been the subject of much speculation and studies [40]. Platelets are believed to mediate inflammatory and other immune processes that drive the progression of kidney disease [41]. The study findings reflect increased platelet activity and, subsequently, the risk of thrombotic events in these PLWD and thus emphasize the need for comprehensive treatment plans.

Hyperfiltration and glomerular hypertrophy mark the initial stages of diabetic nephropathy. This is an adaptive reaction to hyperglycaemia, which also puts podocytes under more mechanical stress and fosters their shedding and subsequent elevation in the urine of PLWD [42, 43]. Growing evidence suggests that podocyte stress and inflammatory signalling play a central role in the pathogenesis of diabetic nephropathy, with enzymes such as dipeptidyl peptidase‐4 (DPP‐4) increasingly recognized as important mediators of these processes. Experimental evidence indicates that enhanced DPP‐4 activity may contribute to oxidative stress, podocyte apoptosis, and structural alterations such as mesangial expansion and albuminuria, all hallmarks of progressive diabetic kidney injury [44].

Therefore, podocyte biomarkers present a more reliable means of predicting and diagnosing early diabetic kidney disease.

As reported in other studies [14, 45, 46, 47], PLWD in this cohort have significantly higher levels of nephrin and podocalyxin. ROC analysis showed that increasing nephrin levels at an AUC of 0.774 and podocalyxin at an AUC of 0.744 are significantly associated with diabetic kidney disease. This result agrees with findings by several other researchers [48, 49, 50, 51]. The presence of nephrin and podocalyxin in urine is an early indicator of damage to the kidneys, even before significant functional decline is indicated, and therefore, a potentially better predictor of renal damage than albuminuria, which will be detectable only after significant damage to the kidneys has occurred.

In this cohort, nephrin emerged as the best biomarker for detecting macroalbuminuria among PLWD with a sensitivity of 76.6%, a specificity of 76.9%, an area under the curve of 77.4% and an accuracy of 76.7. Nephrin is a transmembrane protein that is almost exclusively expressed in glomerular podocytes. Given its greater renal specificity compared with podocalyxin, which can be found in other tissues such as endothelial cells and breast epithelium [52], urinary nephrin may more directly reflect underlying kidney injury. Nevertheless, these findings should be interpreted cautiously, given the limitations of the study.

### 4.1 Study Limitations

This study is limited by the cross‐sectional design, which makes it impractical to establish a causal relationship between the studied biomarkers and kidney disease. This study defined DN based on albuminuria and then tested the podocyte biomarkers against this same marker, thereby creating a circularity problem. This limits the strength of conclusions drawn from comparing the podocyte markers to albuminuria.

Additionally, the ROC‐derived cut‐off values were generated and evaluated within the same dataset, introducing a risk of optimism bias and potentially inflating estimates of diagnostic performance. Accordingly, these thresholds should be regarded as exploratory. Also, internal validation procedures such as bootstrap resampling or split‐sample validation were not performed due to sample size limitations and should be addressed in future studies. However, our findings are consistent with studies by other researchers. Future research should consider using more robust diagnostic criteria, such as a kidney biopsy, to better validate the podocyte markers among Ghanaians.

## 5 Conclusion

Urinary podocyte markers, particularly nephrin, are promising biomarkers that should be validated prospectively in a larger Ghanaian cohort. Nephrin, together with haematological indices, could potentially provide sensitive yet inexpensive biomarkers for early diagnosis of diabetic nephropathy.

## Author Contributions

**Helen Owusu‐Asante:** conceptualization, methodology, data curation, investigation, writing – original draft, validation, resources, project administration. **Samuel Nkansah Darko:** conceptualization, methodology, supervision, writing – review and editing, investigation. **Max Annani‐Akollor:** supervision, visualization, writing – review and editing, investigation. **Ebenezer Senu:** methodology, validation, visualization, software, formal analysis, data curation, writing – review and editing.

## Conflicts of Interest

The authors declare no conflicts of interest.

## Data Availability

Data sharing not applicable to this article as no datasets were generated or analysed during the current study.
